# Universality of ecological memory for local and global net ecosystem exchange, atmospheric CO_2_, and sea surface temperature

**DOI:** 10.1038/s41598-024-73641-z

**Published:** 2024-10-29

**Authors:** Allan Roy B. Elnar, Christopher C. Bernido

**Affiliations:** 1 Department of Physics, University of San Carlos, Talamban, Cebu City, 6000 Philippines; 2 Department of Chemistry and Physics, Cebu Normal University, Cebu City, 6000 Philippines; 3 Research Center for Theoretical Physics, Central Visayan Institute Foundation, Jagna, 6308 Bohol Philippines

**Keywords:** Universality of ecological memory, Atmospheric ${\text {CO}}_{2}$ levels, Global net ecosystem exchange, Global sea surface temperatures, Ecological modelling, Climate and Earth system modelling

## Abstract

Modeling global net ecosystem exchange is essential to understanding and quantifying the complex interactions between the Earth’s terrestrial ecosystems and the atmosphere. Emphasizing the inter-relatedness between the global net ecosystem exchange, global sea surface temperature, and atmospheric ${\text {CO}}_{2}$ levels, intuitively suggests that all three systems may exhibit collective environmental memory. Motivated by this, we explicitly identified a collective memory function and showed a similar non-Markovian stochastic behavior for these systems exhibiting superdiffusive behavior in short time intervals. We obtained the values of the memory parameter, $\mu$, and the characteristic frequencies, $\nu$, for global net ecosystem exchange (GNEE) ($\mu = 0.94\pm 0.03, \nu = 0.67\pm 0.08/mo.$), global sea surface temperature (GSST) ($\mu = 0.68\pm 0.11, \nu = 0.30\pm 0.18/mo.$), and atmospheric ${\text {CO}}_{2}$ ($\mu = 0.78\pm 0.08, \nu =0.66\pm 0.13/wk.$). The values of the memory parameter are within the range, $0< \mu < 1$, and thus all three systems are in the superdiffusive regime. We emphasize, further, that these results were consistent with our previous analyses at the ecosystem level (i.e. Great Barrier Reef) suggesting *scale invariance* for these phenomena. Thus, the observed *superdiffusive* behavior operating at different scales suggests *universality* of the collective memory function for these systems.

## Introduction

Over the past decades, carbon emissions have been at unprecedented levels raising land and ocean surface temperatures that drive extreme weather events and destroy ecological systems. The inevitable effects of these disrupted ecosystems, when uncontrolled, may push these ecosystems to their tipping points and be irreversibly damaged. These rising levels observed in many global environmental systems such as sea surface temperature and anthropogenic ${\text {CO}}_{2}$ emissions have been disrupting the natural cycles of atmospheric events^1–3^. Among the most impacted natural cycles is the *global carbon balance* whereby observed fluctuations are indicative of changing ecosystem behavior, such as microbial regulations of greenhouses fluxes^4^, altered peatland vegetation phenology^5^, shifting of forest ecosystems from sink to source, that may result to the tipping of carbon balance^6^. Significant disruptions can lead to non-linear state shifts and the degree of recovery for these systems relies mostly on their *ecological memory*^7^. While often ignored in many dynamical models of ecosystems and other complex systems, it influences dynamics, composition, and stability landscape as the system evolves to its future state. A system exhibits ecological memory when this future state do not only depend on its current state but also on its initial state and trajectory^8^. The use of this ecological parameter has allowed many to probe the underlying mechanisms of ecosystem interactions and of interrelated environmental systems’ responses and feedback to both natural and anthropogenic disturbances^9,10^.

*Ecological memory* in complex systems, such as those observed in ecosystem models, operates as a response to external influences. For example, biological mechanisms such as acclimatization, adaptation, and shifts in community composition are an ecosystem’s response to the cumulative stress of past disturbances^11,12^. In a similar manner, these external influences, such as climate disturbances, may also be operating with underlying memory, called *climate memory*^13^. In Elnar et al. (2021), interactions between the Great Barrier Reef (GBR), sea surface temperature (SST), and atmospheric ${\text {CO}}_{2}$ levels revealed a *collective ecological memory*. Similarly, this collective ecological memory shaped evolutionary mechanisms such as the observed genetic trait divergence, i.e. genetic memories^14^ and migration dynamics^15^. It follows that systems exhibiting dynamical behavior over time are described with similar *memory* functions, for example the hard coral cover of GBR, SST, and atmospheric ${\text {CO}}_{2}$ as done in Elnar et al. (2021). This exemplifies the dynamical property of *universality*, the tendency of diverse systems to partition themselves under one “universality class”^16^ and these functions have become a powerful tool in predicting and understanding the behavior of these systems^17^. Note that universality of a function is often realized by invariance in scales of length, energy, and time or space as in this present case. Systems exhibiting this property often are considered robust under external influences and better predictability^18^ such as those applied in climate systems, ecology and economics, and other complex systems^16,17,19^.

Moreover, the dynamics of SST and atmospheric ${\text {CO}}_{2}$ operate both at the ecosystem level (i.e. GBR-level^20^) and on the global scale, suggesting *scale invariance*. We demonstrated that the same dynamical behavior of these climatic events shows universality relative to their ecological memory. In addition, we show that interrelated climatic phenomena operate with a collective ecological memory at the global scale for global net ecosystem exchange (GNEE), global sea surface temperature (GSST), and atmospheric ${\text {CO}}_{2}$ using white noise analysis. To show this, we analyzed the generated 10-year monthly *GNEE* dataset of Jiang et al. (2022) using the stochastic framework with memory^32,33^. This framework provides a direct comparison between analytical and empirical results of the mean square displacements (MSDs) of *GNEE*. By using the theoretical MSD for global sea surface temperature (GSST) and atmospheric ${\text {CO}}_{2}$ levels, this is matched to the empirical MSD of *GNEE*. Once the theoretical MSD had been matched with the empirically generated MSD of the fluctuating values of the observables, we obtained an explicit analytical probability density function (PDF) describing the *GNEE*.

## Methods

### Observable fluctuations in empirical datasets

Considering that the global datasets for sea surface temperature, the atmospheric ${\text {CO}}_{2}$, and the global net ecosystem exchange (GNEE) exhibit fluctuating observable, their behaviors were captured following the method on the stochastic framework with memory using white noise analysis^33^ such that the fluctuating variable *x*(*t*) was parameterized as

1 $$\begin{aligned} x(T)&= x_{0} + \int _{0}^{T} (T-t)^{(\mu -1)/2} t^{(\mu - 1)/2}\nonumber \\&\quad \times \sqrt{sin(\nu t)} dB(t) \end{aligned}$$

when these systems evolved in time $t = [0, T] = [0, \pi /\nu ]$. Here, we express the derivative of the Brownian motion as, $dB(t) = \omega (t)dt$, where $\omega (t)$ is a Gaussian white noise random variable. We explicitly used the memory function $f(T-t) = (T-t)^{(\mu -1)/2}$ and the modulating function, $h(t) = t^{(\mu - 1)/2} \sqrt{sin(\nu t)}$, to the Brownian motion (Bernido and Bernido 2015, Table 3.1 Eq. 9. p. 25).

When the endpoints of $x(t = T)$ are fixed to be at $x_{T}$, the probability for this system is of the form

2 $$\begin{aligned}&P(x_{T}, T; x_{0}, 0) = \left( \frac{\pi ^{-\frac{1}{2}} T^{\frac{1}{2} -\mu } \nu ^{\mu -\frac{1}{2}}}{2\Gamma (\mu ) \sin \left( \frac{\nu T}{2}\right) J_{\mu -\frac{1}{2}} \left( \frac{\nu T}{2}\right) }\right) ^{\frac{1}{2}}\nonumber \\&\quad \times \exp \left[ \frac{-T^{\frac{1}{2}-\mu } \nu ^{\mu -\frac{1}{2}} (x_{T} - x_{0})^{2}}{2\sqrt{\pi } \Gamma (\mu ) \sin \left( \frac{\nu T}{2}\right) J_{\mu -\frac{1}{2}}\left( \frac{\nu T}{2}\right) }\right] \end{aligned}$$

From the PDF above, the mean square displacement is evaluated, $MSD = \langle x^2 \rangle - \langle x \rangle ^2$, and is obtained as

3 $$\begin{aligned} MSD = \frac{\Gamma (\mu ) \sin (\nu T/2) J_{\mu - \frac{1}{2}} (\nu T/2)}{\pi ^{-\frac{1}{2}} T^{\frac{1}{2} - \mu } \nu ^{\mu - \frac{1}{2}}} \end{aligned}$$

where $\Gamma (\mu )$ is the gamma function and $J_{\mu }$ is the Bessel function of the first kind, as before.

### Fluctuating observables for global sea surface temperature

**Fig. 1:**
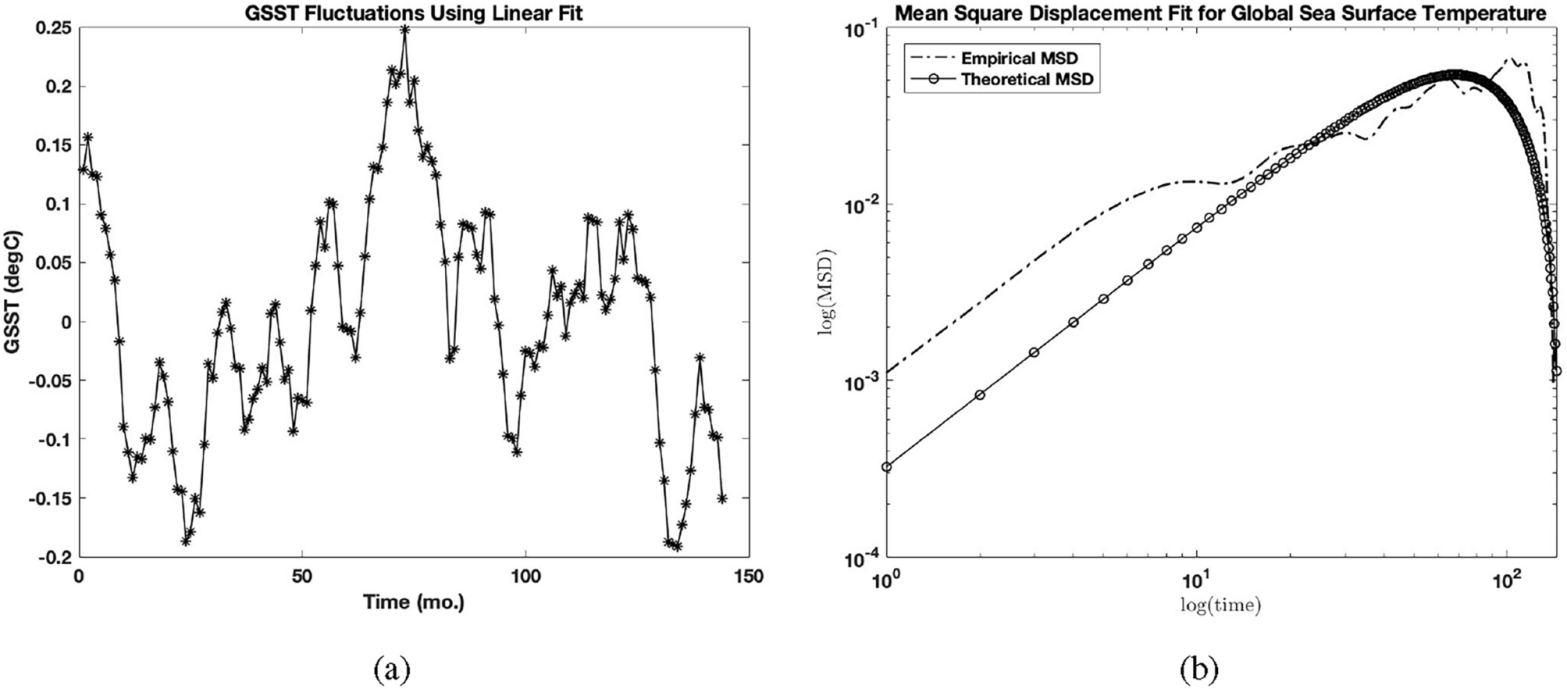
(**a**) Global sea surface temperature fluctuations from January 2010 to December 2021. (**b**) Empirical GSST MSD (dash line) and theoretical MSD (line-circle) match ($r^2 = 0.6538$) for $\mu = 0.68$, $\nu = 0.335$, the x- and y- shift of $t_{c} = 9.18$ and $N=0.0198$, respectively.

The monthly global sea surface temperature (GSST) datasets obtained from January 2010 to December 2021 (https://www.ncei.noaa.gov/access/monitoring/climate-at-a-glance/global/time-series/globe/ocean/all/1/2010-2021) were investigated instead of the localized time-series of Niño 4 near the Great Barrier Reef as done in Elnar et al. (2021). GSST fluctuations using a linear fit are shown in Fig. 1a.

### Fluctuating observables for atmospheric ${\text {CO}}_{2}$ level

**Fig. 2:**
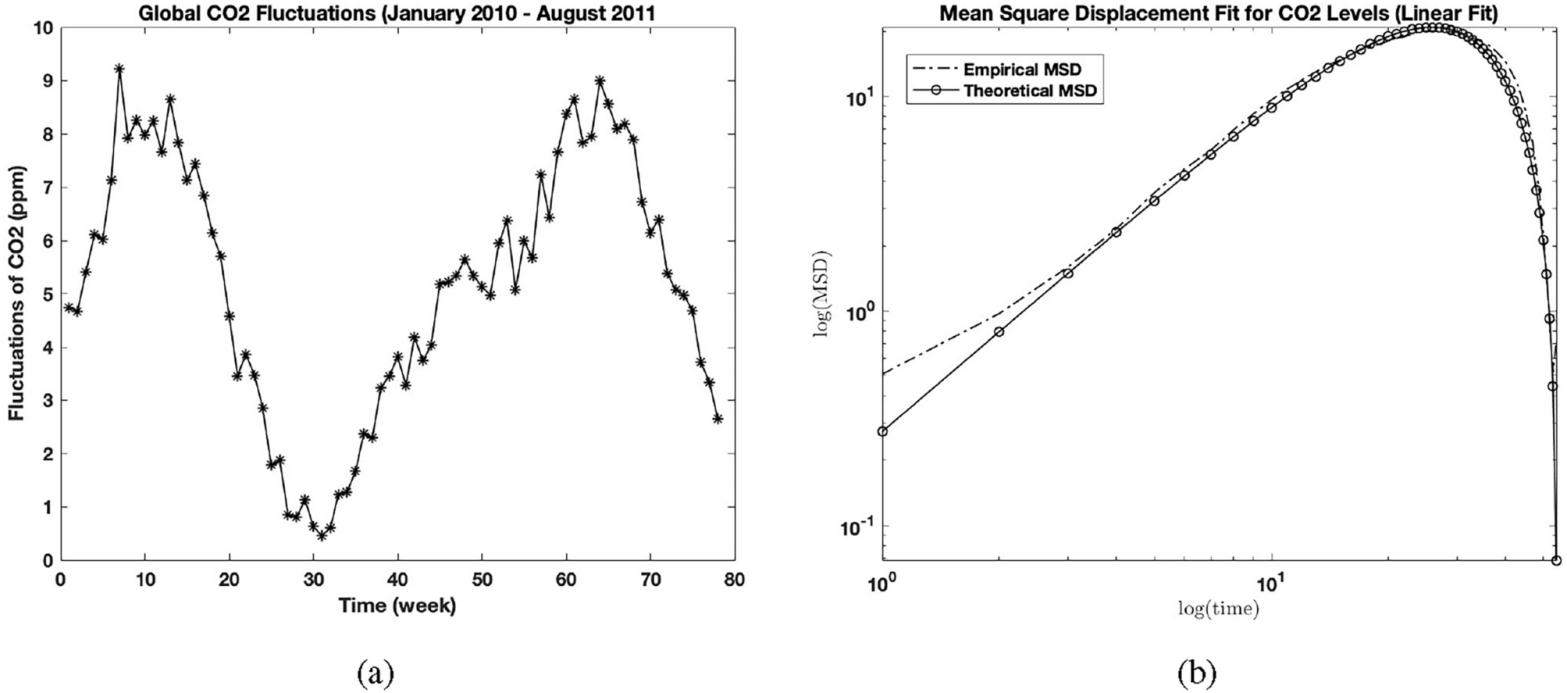
(**a**) Global $${\text {CO}}_{2}$$ fluctuations from January 2010 to August 2011. (**b**) Empirical linear fitted atmospheric $${\text {CO}}_{2}$$ level MSD (dash line) and theoretical MSD (line-circle) match ($$r^2 = 6561$$) for $$\mu = 0.78$$, $$\nu = 0.66$$, $$t_{c} = 6.349$$$$N=9.079$$.

Atmospheric ${\text {CO}}_{2}$ level weekly dataset from the the Mauna Loa Observatory of the National Oceanic Atmospheric Administration (NOAA, www.esrl.noaa.gov) was obtained from the Keeling curve from January 2010 - August 2011. We have generated the corresponding fluctuations of the observables from this dataset as shown in Fig. 2a as done in Elnar et al. (2021). From the Keeling curve, the fluctuations were obtained using a linear fit.

### Assimilated global net ecosystem exchange (GNEE)

#### Datasets

**Figure 3:**
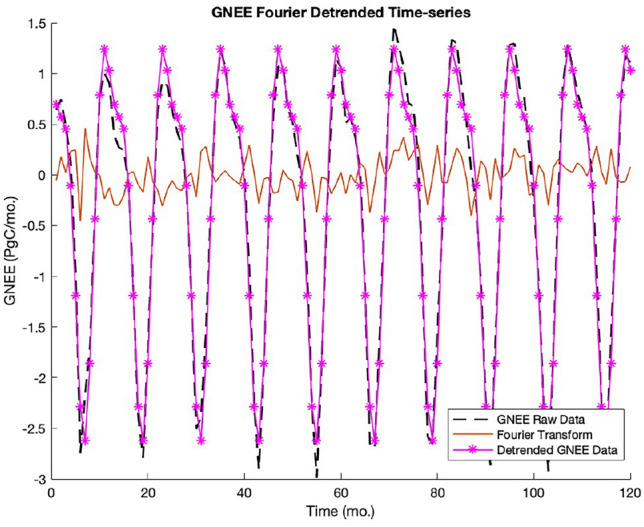
Plot of the original GNEE (black dash line), filtered signal via Fourier transform (solid orange line, and the detrended GNEE data (line asterisk (purple line)).

We investigated the assimilated global surface carbon fluxes by Jiang et al. (2022) inferred using a *global carbon assimilation system v2* (GCASv2). This is a machine learning algorithm that estimates surface carbon fluxes using satellite $XCO_{2}$ retrievals. The machine learning system used is composed of three algorithms, namely the model for ozone and related chemical tracers, version 4 (MOZART-4)^54^ that is coupled to a 3-D atmospheric ${\text {CO}}_{2}$ concentration simulator and ensemble square root filter (EnSRF) implemented via inversion of surface fluxes^38,39^. The assimilated GNEE is a 10-year monthly dataset from January 2010 to December 2019 or a total of 120 data points and its detailed description is retrievable at zenodo.org (https://doi.org/10.5281/zenodo.5829774) shown in Fig. 3. It was described herein that carbon flux measurements were obtained from fluxes of biological origin, ocean fluxes, fossils, and fire^37^.

#### Detrending method

Since, we are interested in the stochastic part of the dataset rather than their dominant part, we employed the Fourier Transform methods. To note, as in many applications, detrending datasets reveal seasonality, time-lags and other related information by removing noise in the dataset. However, in this framework, we are interested in the analysis of the noise part of the datasets.

*Fourier Transform (FT) Method* For further comparison, a Fourier transform was employed on the time series dataset for the same purpose of detrending GNEE. There is a vast literature on employing the Fourier transform in the spectral analyses of time series data. Accordingly, recent studies used this method to detrend variability of ${\text {CO}}_{2}$ on tropical forests^44^ and global shifts in vegetation resilience^45^. Briefly, the time-series dataset of GNEE was converted to the frequency domain via the Fourier transform, removed data points that make spikes in the transform, and returned to its original format by taking the inverse Fourier Transform. The transformed GNEE, the filtered signal, and the original GNEE dataset are presented in Fig. 5.

## Results

### Fluctuating observables for global sea surface temperature

Evaluation of the mean square displacement of GSST fluctuations in Fig. 1a yields an MSD shown in Fig. 1b. The empirical and theoretical MSDs matched ($r^2 = 0.6538$) for parameter values change for $\mu$ between 0.57 and 0.79 and for $\nu$ between 0.12 and 0.48/mo. The analytical MSD was adjusted without altering its shape using a normalization *N*(MSD), where $N = 0.0198 \pm 0.001$ and the time variable as $t/t_{c}$ where $t_{c} = 9.18 \pm 4.76$.

### Fluctuating observables for atmospheric ${\text {CO}}_{2}$ level

Evaluating the fluctuations for the atmospheric ${\text {CO}}_{2}$ levels in Fig. 2a using Eq. (3) yields a good match ($r^2 = 0.6561$) of the empirical and analytical MSDs as shown in Fig. 2b as done in Elnar et al (2021). The parameter values obtained for $\mu$ is between 0.70 and 0.86 and $\nu$ between 0.53 and 0.79/wk. Again, the analytical MSD was adjusted without altering its shape using a normalization *N*(MSD), where $N = 9.08 \pm 0.66$. and the time variable as $t/t_{c}$ where $t_{c} = 6.35 \pm 1.18$.

Note that a good match between empirical and theoretical MSDs for both the global sea surface temperature and the atmospheric ${\text {CO}}_{2}$ level were obtained. With the close relationship of the global net ecosystem exchange (GNEE), it is shown herein that it too can be evaluated and described with the same stochastic memory function.

### Empirical versus theoretical MSD’s of Fourier transform (FT) detrended GNEE

**Figure 4:**
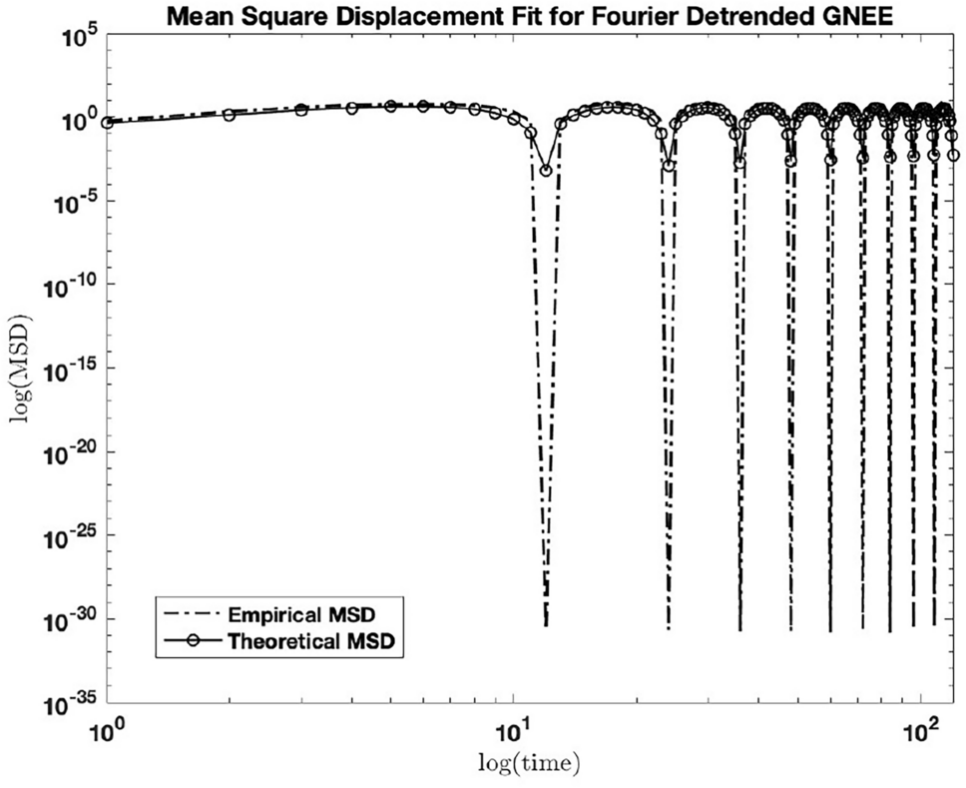
Empirical vs. theoretical MSD for FT detrended GNEE. matched ($$r^2 = 0.905)$$ for $$\mu = 0.94$$, $$\nu = 0.67$$, $$t_{c} = 1.273$$, and $$N = 2.629$$.

The MSD of the FT detrended GNEE data in Fig. 3 had a good match ($r^2 = 0.905$) compared to the theoretical MSD of Eq. (3). The parameter values obtained for $\mu$ is between 0.91 and 0.97, for $\nu$ is between 0.59 and 0.75/mo, and the x- and y- shifts of $t_{c} = 1.273 \pm 0.073$, $N = 2.629 \pm 0.169$. The matched empirical and theoretical MSDs of the FT-detrended GNEE are shown in Fig. 4 below.

We emphasize that the parameter estimates were obtained with 95% confidence and the matched empirical and theoretical MSD’s for all systems revealed a relatively high correlation values: GNEE ($r^2 = 0.905$), GSST ($r^2 = 0.6538$), and atmospheric ${\text {CO}}_{2}$ ($r^2 = 0.6561$). Without altering the shapes of the empirical MSD’s, they are captured by the same analytical MSD in Eq. (3), hence these systems can be described with a stochastic memory function collectively. To gain further insights, the PDF’s associated with the MSD in Eq. (3) were obtained and plotted for different time length as described in the following section.

### Probability density function (PDF) for GNEE, GSST, and atmospheric CO_2_

**Figure 5:**
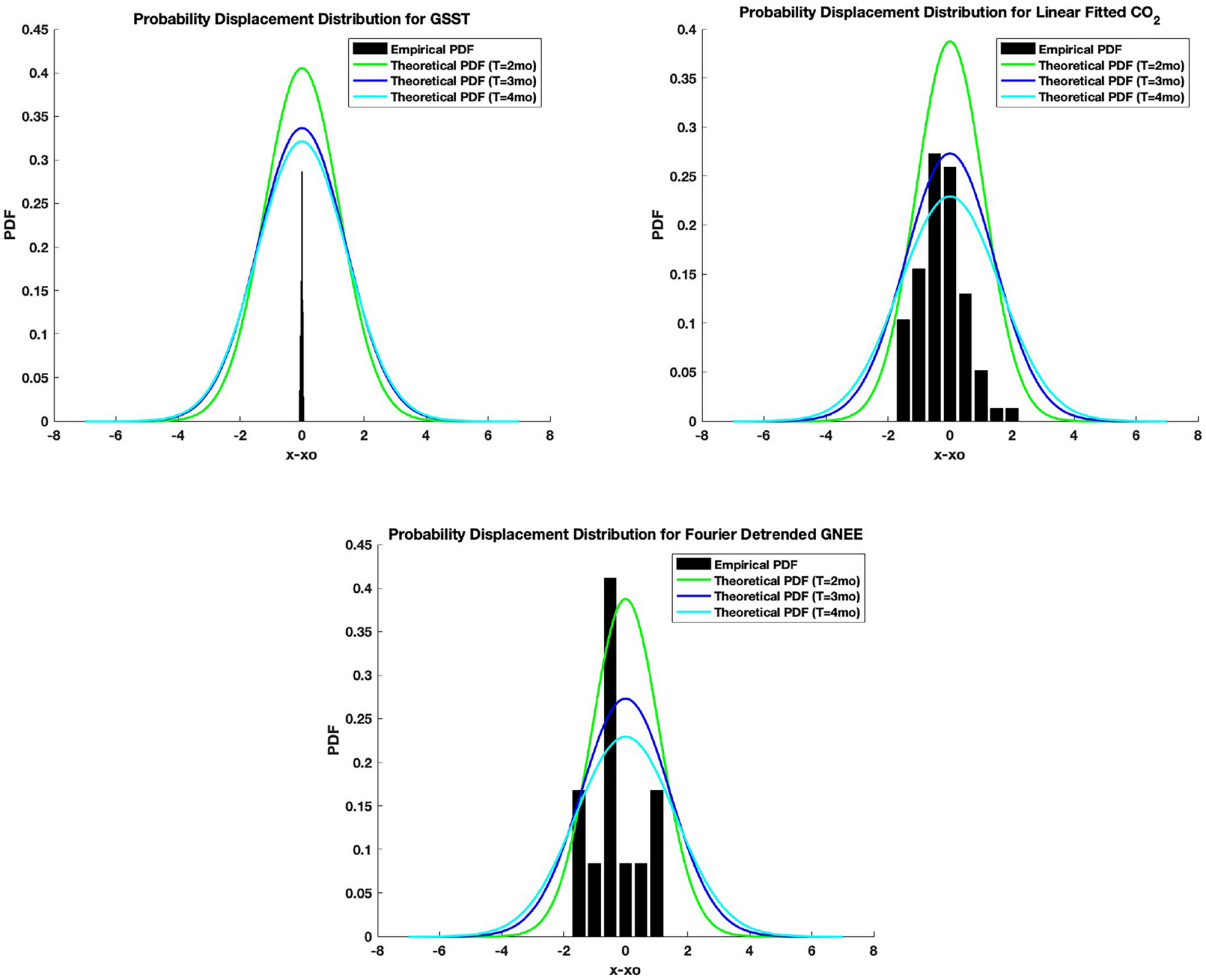
Probability displacement distributions for (**a**) *Linear* fitted Global sea surface temperature; (**b**) *Linear* fitted ${\text {CO}}_{2}$; (**c**) *Fourier* transformed GNEE.

The probability density function obtained in Eq. (2) given the memory function $(T-t)^{(\mu - 1)/2}$ can be tested against the empirical datasets as shown in Fig. 5. With the values of $T = (2, 3, 4)$, one gains insights in the respective probability displacement distribution (PDD) of the systems using the memory parameter, $\mu$, and characteristic frequency, $\nu$ in Table 1. The peak of PDFs decreases as the displacement in time increases. Consistently, the corresponding PDFs for each model reflect reduced probabilities at longer times.

**Table 1:** Values for the memory parameter $\mu$ and characteristic frequency $\nu$ for GNEE, GSST, and ${\text {CO}}_{2}$ levels.

System	Memory	Characteristic
	parameter, $\mu$	frequency, $\nu$
GNEE	$\mu =0.94 \pm 0.03$	$\nu =0.67 \pm 0.08$ /mo.
$CO_{2}$ levels	$\mu =0.78\pm 0.08$	$\nu =0.66 \pm 0.13$/wk.
Global SST	$\mu =0.68 \pm 0.11$	$\nu =0.3 \pm 0.18$/mo.

## Discussion

The close relationship of global net ecosystem exchange (GNEE) to sea surface temperature and ${\text {CO}}_{2}$ levels had been elucidated in the reports of ${\text {CO}}_{2}$ fluxes in semi-arid regions^46,47^, land-atmosphere ${\text {CO}}_{2}$ exchanges^48^?, and/or in estuarine ecosystems^21,49^. To help understand their interrelated dynamics we used an analytic framework for stochastic processes with memory as implemented in empirical models of ecological and environmental systems describing *GBR*, *SST*, and ${\text {CO}}_{2}$ levels^20^ and cyclone track dynamics^33^, dynamics of ageing fibrin^34^, DNA distributions in genomes^35^, and diffusion coefficients of proteins with varying lengths^36^, among others. We have shown here that a good match between empirical and theoretical MSDs was attained for each phenomenon indicating that all three systems are described by the same class of non-Markovian stochastic processes described by Eqs. (2) and (3). Summarily, Table 1 below presents the values of the memory parameter $\mu$ and characteristic frequency $\nu$ where comparative insights into the complex dynamics of GNEE, GSST, and atmospheric ${\text {CO}}_{2}$ levels can be seen. For both the memory parameter, $\mu$, and characteristic frequency, $\nu$, GSST and atmospheric ${\text {CO}}_{2}$ have relatively the same values while GNEE registers the highest value. It is reiterated that despite these systems being influenced by the same memory behavior, their memory functions differ in values of $\mu = 0.94$ for GNEE, $\mu = 0.78$ for atmospheric ${\text {CO}}_{2}$, and $\mu = 0.68$ for GSST.

**Figure 6:**
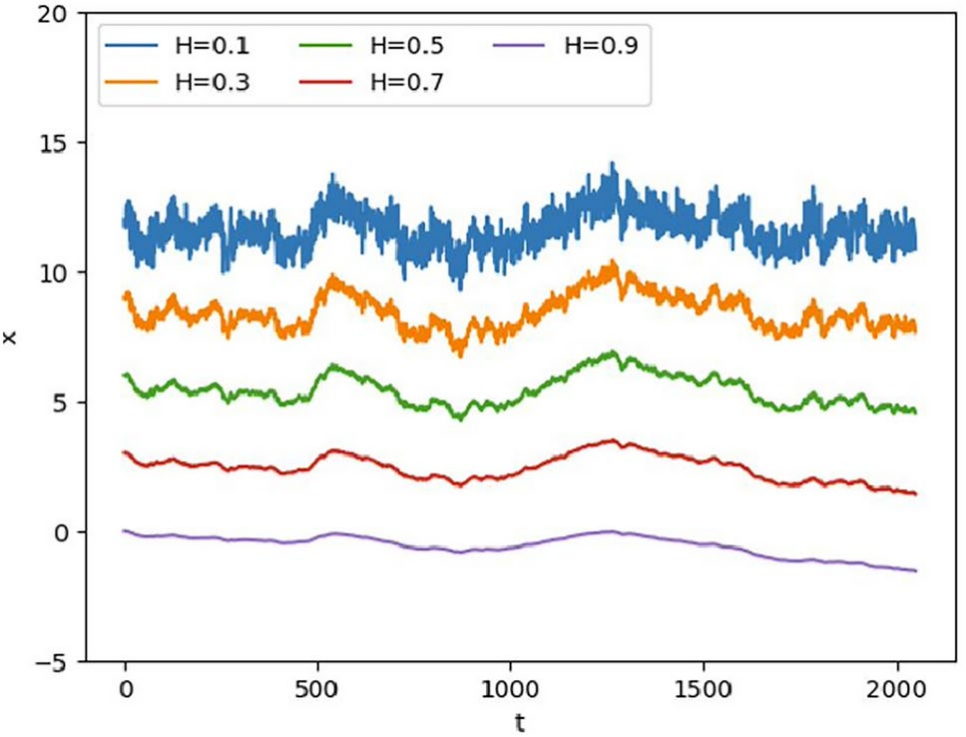
Paths of fractional Brownian motion for different values of the Hurst exponent *H*. Subdiffusive paths have, $0< H < 0.5$. Superdiffusive paths have $0.5< H < 1$. Only ordinary Brownian motion ($H = 0.5$), has no memory. Top to bottom: $H = 0.1$, $H = 0.3$, $H = 0.5$, $H = 0.7$, and $H = 0.9$.

For short time interval analyses, $$T<< 1$$, the asymptotic forms of $$\sin (\nu T/2) \approx \nu T/2$$ and $$J_{\mu -1/2} (\nu T/2) \simeq \left[ 1/\Gamma (\mu + 1/2)\right]$$$$(\nu T/4)^{\mu -1/2}$$ was used which reduces Eq. (3) to an MSD exhibiting a power law such that

4 $$\begin{aligned} MSD \approx bT^{\beta } \end{aligned}$$

where, $\beta = 2\mu$, and $b = \frac{\sqrt{\pi }\ \nu \Gamma (\mu )}{4^{\mu } \Gamma (\mu +1/2)}$. As for the three systems described, their corresponding $\beta$ values are $\beta = 1.88$ for GNEE, $\beta = 1.56$ for atmospheric ${\text {CO}}_{2}$, and $\beta = 1.36$ for GSST. The physical implication of these values indicates that GNEE, atmospheric ${\text {CO}}_{2}$, and GSST exhibit *superdiffusive* behavior, i.e. $1< \beta < 2$, in their growth of MSD. For clarity of the superdiffusive behavior, we note that many physical systems have observables that fluctuate that one can contrast fluctuations with or without memory as exemplified by fractional Brownian motion shown in Fig. 6. The value of the Hurst exponent H characterizes the memory behavior where we can numerically simulate the fractional Brownian motion. This is done using the Wood-Chan or circulant method for paths of the fractional Brownian motion.^58^. To illustrate, for, $H = 0.5$, the fluctuations exhibit ordinary Brownian motion with no memory. In contrast, the fluctuations for, $H \ne 0.5$, exhibit memory behavior and are non-Markovian. In particular, $H < 0.5$, shows subdiffusive fluctuations where neighboring datapoints are anti-correlated. On the other hand, $H > 0.5$, are referred to as superdiffusive fluctuations where neighboring datapoints are positively correlated (see, e.g., Fig. 6).

The results in the present analyses for GNEE, GSST, and atmospheric ${\text {CO}}_{2}$ levels are consistent with the collective memory function for GBR, SST, and atmospheric ${\text {CO}}_{2}$ levels^20^ at the ecosystem level for short time interval (e.g. local vs. global scale)^50^. Hence, the superdiffusive behavior of these systems is *scale invariant*. The latter corroborated the dynamical observations on the reported estimates for net ecosystem exchange (NEE) at the regional and global scales^51^, across space^52^ and time^53,55^ despite the spatio-temporal heterogeneity in the drivers of ${\text {CO}}_{2}$ fluxes^56,57^. Note that, we have viewed three complex systems (or even more): the GNEE, GSST, and atmospheric ${\text {CO}}_{2}$ through a common lens in a unified stochastic process with memory. This comprehensive framework enables us to compare the patterns shown of the data sets generated by each system. The analysis on the behavior of the data sets through a stochastic framework reveals that all three systems have characteristics of long-term memory. For short time intervals, $T<< 1$, equation (4) indicates that the mean square displacement of the observable grows over time as $MSD \approx bT^{\beta }$, where $\beta =2\mu$ and *b* depends on the memory parameter $\mu$ (see Table 1). Specifically, the exponents for all three systems are superdiffusive, with values between $1< \beta < 2$. To note, our previous study revealed similar superdiffusive behavior for these phenomena at the ecosystem level (e.g. GBR-level^20^). Consistent with reported estimates for net ecosystems exchange, ${\text {CO}}_{2}$ uptake, and temperature^51–53^, the dynamics of these phenomena are considered *scale invariant*. Thus, observed *superdiffusive* behavior at different scales suggest *universality* of the collective memory function for these systems. As described above, the universality of the memory function is robust and improves predictability^18^ much more of its inclusion in the analyses of their dynamics^23,24,59^ such as dryland global carbon cycle contribution using the global terrestrial biosphere model including those predicted for urban forests^25,26^, peatland mosses^5^, tropical vegetation^27^, as well as environmental processes of soil respiration^28,29^ and land-ocean-atmosphere carbon exchange^22,30,31^.

**Figure 7:**
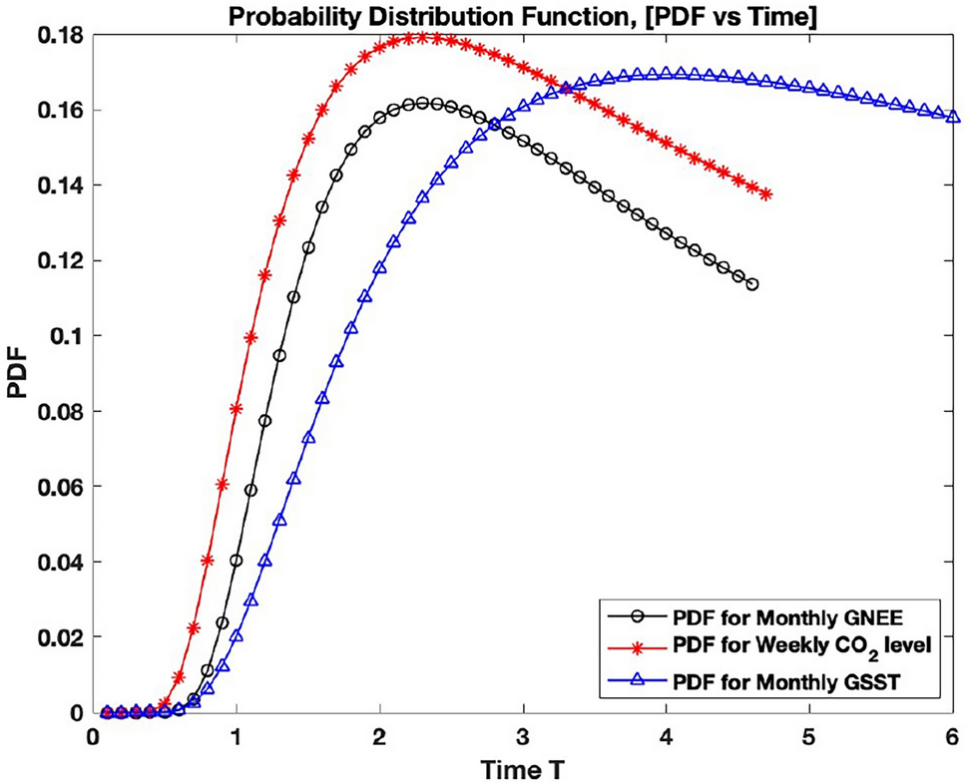
Probability distribution for GNEE (black line-circle), GSST (blue line-triangle), and atmospheric ${\text {CO}}_{2}$ (red line-asterisk).

Finally, the evolution in time for fluctuations can be compared for these systems by plotting their PDF against time as illustrated in Fig. 7. The displacement intervals of $\Delta x = 1.5$ for fluctuations were reasonably chosen for the three systems. The bias of these values can be explained given that the average GNEE is within the average inter-annual amplitude^37^, and the global consensus on the temperature increase limit is at 1.5 $^{\circ }$C^53^. Using these values in Eq. (4) and the corresponding memory parameters and characteristic frequencies in Table 1, the PDF of GNEE and atmospheric ${\text {CO}}_{2}$ reaches their peaks roughly around the same time as shown in Fig. 7. What is depicted here describes how fast these systems reached the prescribed threshold of $\Delta x = 1.5$ for the first time, often called *first-passage time (FPT)*^60,61^. Essentially, all three systems have the same probability distributions that slowly diverge at longer times. These scenarios imply that a difference in fluctuations of 1.5 PgC/mo., 1.5 ppm, and $1.5^{\circ } C$ for GNEE, atmospheric ${\text {CO}}_{2}$, respectively, are possible and sustained even for longer times. The MSD in Eq. (3) which is uniquely identified by the memory parameter $\mu$ and the characteristic frequency $\nu$ for each system (as listed in Table 1), we derived an explicit form of the probability density function. This derived probability density function (PDF) gives a new perspective on the temporal interconnections of the global net ecosystem exchange (GNEE), global sea surface temperatures (GSST), and atmospheric ${\text {CO}}_{2}$ levels, providing a mathematical approach to understanding the impact of collective ecological memory.

## Data Availability

Data and MatLab codes used in the manuscript can be accessed through: 10.6084/m9.figshare.25451413.
